# The Reinforcing Effect of Waste Corrugated Paper Fiber on Polylactic Acid

**DOI:** 10.3390/polym14173562

**Published:** 2022-08-29

**Authors:** Jian Su, Zhiwei Jiang, Changqing Fang, Yamin Zheng, Mannan Yang, Lu Pei, Zhigang Huang

**Affiliations:** 1 Faculty of Printing, Packaging Engineering and Digital Media Technology, Xi’an University of Technology, Xi’an 710054, China; 2 School of Mechanical and Precision Instrument Engineering, Xi’an University of Technology, Xi’an 710048, China; 3 Key Laboratory of Processing and Quality Evaluation Technology of Green Plastics of China National Light Industry Council, Beijing Technology and Business University, Beijing 100048, China

**Keywords:** waste corrugated paper fiber, recycle, polylactic acid, composite material, reinforce

## Abstract

To improve the recycle value of waste paper and promote circular economic development, waste corrugated paper fiber (WCPF) was used as a reinforcing agent to prepare waste corrugated paper fiber/polylactic acid (WCPF/PLA) composites via dichloromethane solvent which can be reused. The WCPF in the waste corrugated paper is extracted by beating in a Valli beating machine for different time lengths and grinding in a disc grinder. The effects of beating time and the content of WCPF on the microstructure, mechanical properties, thermal decomposition process, and crystallization properties of the WCPF/PLA composite were studied. The result shows that the WCPF can be well separated from each other and can be evenly dispersed in the PLA matrix. When 25 wt% WCPF which was beat for 30 min was used, the composite has the greatest improvement in tensile property. This study provides a new process for the recycling of waste paper in the application of polymer reinforcement. The research on waste paper fiber and degradable polymer composite is of great significance for reducing environmental pollutants and developing circular economy.

## 1. Introduction

The corrugated box is one of the main packaging products used in the field of production and daily life because it is environmentally friendly and has a wide source of raw materials. Generally, the raw materials of corrugated paper contain 9–31 wt% softwood and 62–90 wt% hardwood along with a high lignin content. The core paper is normally made of chemical hardwood pulp with an addition of no more than 15 wt% long fiber coniferous pulp [1]. At present, the recovery technology of corrugated box is relatively single, it is mainly used for the manufacture of recycled paper and molded pulp [2]. In addition, there are also some reports on the preparation of carbon microspheres, porous carbon, and cellulose nanocrystals, etc., from waste paper, but the product yield is low [3,4,5]. With the increasing awareness of environmental protection, biodegradable polymer materials have been paid more and more attention. However, the high cost and some property defects of most biodegradable polymers make it difficult to completely replace the traditional polyolefin materials. Plant fiber is a good material for the reinforcement of degradable polymers because of its wide sources and environmental friendliness [6]. Among the various degradable polymers, the synthesis technology of PLA is more mature, and it can be mass-produced. PLA has a good processing formability and can be processed by extrusion and blow molding. PLA is mainly used in 3D printing, agricultural mulch film, packaging material, and other fields [7]. Although PLA has good elasticity, high rigidity, and excellent thermal formability, etc., the disadvantages such as poor toughness, brittleness, and poor hydrophobicity still limit its application [8,9]. Therefore, many researchers focus on improving its mechanical properties.

Filling modification is a physical modification by adding inorganic or organic fillers to improve the performance of the target product and reduce the cost of raw materials [10,11,12]. Some researchers have indicated that PLA can be enhanced by plant fiber, carbon fiber, and glass fiber, etc. The reinforcement of PLA with plant fiber can not only reduce the preparation cost, but also improve the strength and stiffness of PLA. In addition, plant fibers have the characteristics of low density, good biodegradability, and high safety [13].

Plant fiber is a single fiber bundle and mainly contains cellulose, hemicellulose, lignin, and a small amount of silicate and is widely used in composite materials. Couture et al. [14] prepared two kinds of unidirectional flax composites based on PLA. The results showed that the tensile property of flax paper/PLA composite was like that of the glass fiber fabric-impregnated epoxy resin, and the impact strength was higher than that of unreinforced resin. Bartos et al. [15] prepared PLA/sugarcane bagasse fiber composite by injection molding from two kinds of fiber with different fiber characteristics. The results showed that sugarcane bagasse fibers obviously increase the stiffness of PLA, resulting in almost constant tensile strength and increase of impact resistance. Faludi et al. [16] used wood fibers with a large aspect ratio as raw materials to prepare PLA composites by internal mixing method, and the fiber content varied from 0–60% volume percentage. Moreover, bamboo fiber, flax fiber, sisal fiber, hemp fiber, and other plant fibers were also used as raw materials to prepare PLA composites [17,18,19]. Compared with inorganic fillers, plant fiber has the advantages of biodegradability, environmental friendliness, low cost, non-toxicity, and high tensile property in processing [20].

Corrugated paper contains a lot of plant fiber, and the printing of corrugated paper is generally simple, and the adhesive and ink can be easily removed. It is possible to extract plant fiber from waste paper and use it for polymer reinforcement. Zhang et al. [21] prepared PLA/office waste paper fiber composites, and the result shows that the PLA/office waste paper fiber composite has a better performance than PLA/wheat straw fiber composite and PLA/bamboo fiber composite. In this work, the waste office paper was cut into a size of 20 × 10 mm and was directly pulverized through a high-speed pulverizer, then blended with PLA using an open mill. Lei et al. [22] used waste natural cellulosic fibers from waste office paper to reinforce polyurethane elastomer. The waste paper was pulverized directly through a high-speed pulverizer after removing the ink and the composite materials were prepared by in situ polymerization.

Therefore, using the waste paper as a raw material to reinforce PLA has a good prospect. As is known, the main components of waste paper fiber are cellulose, hemicellulose, and lignin, and there are large hydroxyl groups on the surface, which can easily cause agglomeration. The plant fibers in waste paper cannot be well separated from each other by the direct pulverization method through a high-speed pulverizer and the blade of the pulverizer may cut through the fibers. In addition, the melting and blending process will also lead to aging and degradation of PLA molecules. Therefore, the pre-treatment process of waste paper and the blending process of waste paper fiber and PLA still need to be optimized.

In this work, the WCPF from the waste corrugated paper is extracted by beating with a Valli beating machine and grinding in a disc grinder, so the fibers can be better separated from each other. The WCPF and PLA were blended through dichloromethane solvent to avoid the aging of PLA during melt blending, and the dichloromethane solvent can be reused by evaporation and collection. This study provides a new process for the recycling of waste paper in the application of polymer reinforcement, which has positive environmental benefits.

## 2. Materials and Methods

### 2.1. Materials

Polylactic acid (1.246 g/cm^3^, 100 mesh powder, the melt flow index is 8 g/10min (190 °C, 2.16 kg)) used in this study came from Dongguan Yingsheng Plastic Chemical Co., Ltd., Dongguan, China. Dichloromethane (CH_2_Cl_2_, analytically pure) was purchased from Nanjing Chemical Reagent Co., Ltd. (Nanjing, China). Corrugated paper comes from waste corrugated boxes used for express delivery (Xi’an, China). The cellulose, hemicellulose, and lignin contents of WCPF were determined via the Van Soest detergent method [23]. The ethylenediaminetetraacetic acid disodium salt (C_10_H_14_N_2_Na_2_O_8_), sodium borate (Na_2_B_4_O_7_), ethylene glycol ether (C_4_H_10_O_2_), disodium hydrogen phosphate (NaH_2_PO_4_), cetane trimethyl ammonium bromide (C_19_H_42_BrN), and sodium alkyl sulfate (C_12_H_25_SO_4_Na) used for the Van Soest detergent were all analytically pure and were purchased from Nanjing Reagent Co., Ltd. (Nanjing, China).

### 2.2. Preparation of WCPF/PLA Composite

**Figure 1:**
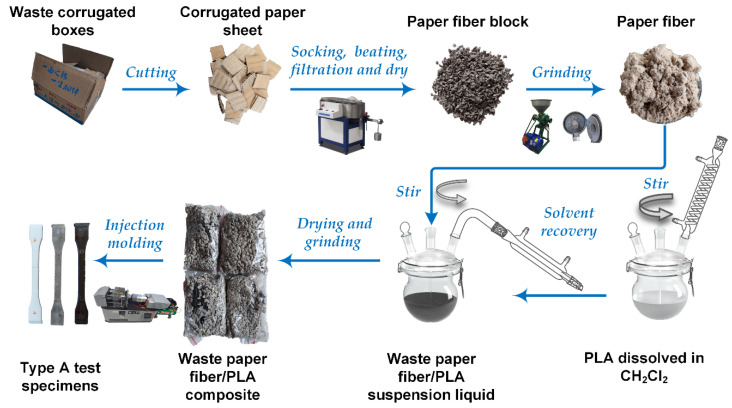
Preparation process diagram of the WCPF/PLA composite.

Figure 1 shows the preparation process of the WCPF/PLA composite. The attachments such as adhesive tape and labels on the waste corrugated boxes were removed first, then the corrugated paper was cut into sheets with a size of about 2 × 2 cm. 600 g of paper sheets were soaked in water for 24 h at room temperature, then pulped for different time lengths (10, 20, 30, 40, 50, and 60 min) in a Valli beating machine (IMT-VL01, Dongguan International material Tester Precision Instrument Co., Ltd., Dongguan, China). The pulp was washed by water and filtered by filter cloth, then dried, and the paper fiber blocks were obtained. The paper fiber blocks were grinded by a disc grinding machine and became separated fluffy paper fiber.

The PLA was dissolved in 640 mL of dichloromethane in a glass reactor at 30 °C in a water bath for 4 h, and the glass reactor was equipped with a condensing device to prevent solvent loss. After the PLA completely dissolved, WCPF was added into the reactor and stirred for 1 h to disperse. The total weight of PLA and WCPF was 200 g. The mass ratios and beating times of WCPF for each sample were determined according to the literature and our previous research, respectively, and were shown in Table 1. After the WCPF was completely dispersed, the temperature of the water bath was raised to 45 °C, and the dichloromethane was volatilized and collected for reuse. Then, the product was dried at 60 °C for 12 h to thoroughly remove the residual solvent and the WCPF/PLA composite was obtained. The obtained WCPF/PLA composite has a low compactness structure and very poor mechanical property, which can be directly grinded into granular form with a size of about 5 × 5 mm by hand.

Type A multipurpose test specimens as specified in ISO 3167 were used for the tensile strength and flexural strength tests and were prepared by a horizontal injection molding machine (TY-7003, Jiangsu Tianyuan Testing Equipment Co., Ltd., Yangzhou, China). The temperatures of the three zones of heating of the injection molding machine were 140, 150, and 160 °C, respectively, and the mold is not heated. The injection pressure was 57 MPa. The injection time, pressure holding time, and cooling time were 5 s, 15 s, and 15 s, respectively.

### 2.3. Characterization

Tensile strength and flexural strength were tested by a universal testing machine (XWW-20A, Shanghai Jiezhun Instruments Co., Ltd., Shanghai, China) according to ISO 527-2 and ISO 178 international standard, and the results were the average of three measurements and the standard deviations were shown in the results. A sensor with a range of 0–10 kN was used. The tensile strength test was carried out with a clamping distance of 115 mm and a tensile speed of 5 mm/min. The flexural strength test was carried out with a pressing speed of 5 mm/min and a specimen span of 64 mm using the three-point flexural method.

Scanning electron microscopy (SEM, SU8010, Hitachi, Tokyo, Japan) was used to analyze the morphology of the WCPF/PLA composite under an acceleration voltage of 3.0 kV. The samples were brittle fractured by freezing with liquid nitrogen and were treated with gold spraying.

The thermal properties of the WCPF/PLA composite were studied by differential scanning calorimetry (DSC, 200F3, NETZSCH-Gerätebau GmbH, Selb, Germany). The thermal history was eliminated with a heating rate of 10 °C/min from 20 °C to 150 °C, and then cooled to 20 °C with a cooling rate of 10 °C/min. Finally, the test was carried out with a heating rate of 10 °C/min from 20 °C to 220 °C. About 10 mg of the sample was used and the test was carried out in the nitrogen atmosphere with a nitrogen flow rate of 50 mL/min.

The thermal stability of the WCPF/PLA composite was analyzed by a thermogravimetric analyzer (TG, 209F3, NETZSCH-Gerätebau GmbH, Selb, Germany) at a heating rate of 10 °C/min from 30 °C to 600 °C. About 10 mg of the sample was used and the test was carried out in the nitrogen atmosphere with a nitrogen flow rate of 50 mL/min.

The crystallization of the WCPF/PLA composite was analyzed by an X-ray diffraction apparatus (XRD, XRD-7000, Shimadzu, Kyoto, Japan) with a scanning speed of 10°/min and a sampling pitch of 0.02° from 5–60°. The test was carried out with Cu target, Kα1 line, Ni filter, 40 kV of voltage, and 50 mA of current.

The functional groups of WCPF were characterized by Fourier transform infrared spectrometer (FTIR, FTIR-8400S, Shimadzu, Kyoto, Japan) using KBr pellets as the sample matrix by transmittance mode. The frequency range was 1000–4000 cm^−1^ and the number of scans was 32 times·s^−1^.

## 3. Results and Discussion

### 3.1. Composition and Morphology Analysis of WCPF

**Figure 2:**
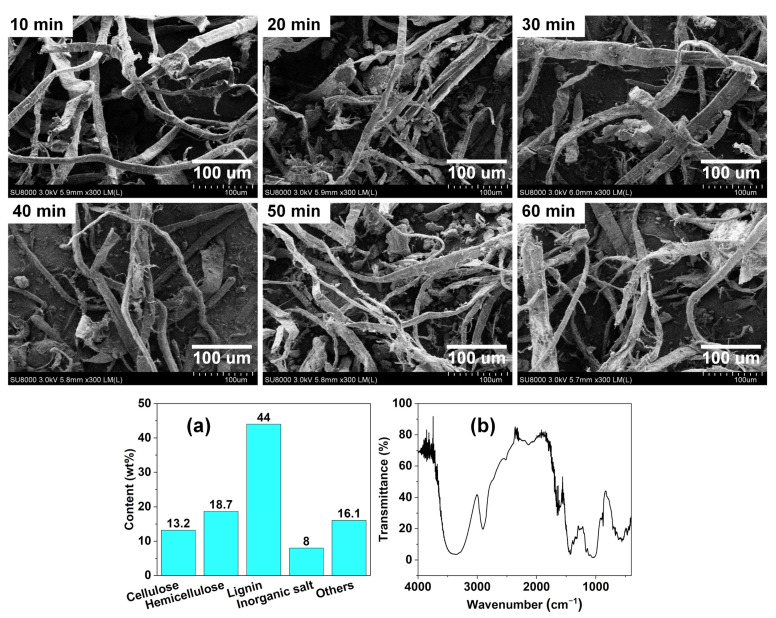
Micromorphology of WCPF with different beating times, composition contents (**a**) and infrared spectrum (**b**) of WCPF.

Figure 2 shows the micromorphology of WCPF with different beating times as well as the composition contents (a) and infrared spectrum (b) of WCPF. The main component of WCPF is lignin, followed by hemicellulose, cellulose, inorganic salts, and other substances. In the infrared spectrum of WCPF, the transmission peak with larger intensity and wider amplitude located at 3500–3300 cm^−1^ is caused by the stretching vibration of intramolecular hydroxyl group (-OH) in cellulose. The transmission peak located at 2940–2855 cm^−1^ is caused by the stretching vibration of -CH, -CH_2_, and intermolecular hydroxyl group (-OH). The transmission peak at 1045 cm^−1^ is caused by the stretching vibration of C=O in cellulose. The transmission peaks located at 640–1445 cm^−1^ and 878 cm^−1^ are caused by the stretching vibration of C=C in the benzene ring skeleton of lignin and the bending vibration of benzene derivatives out of plane, respectively.

The WCPF are mostly flat coarse strips with a large length to diameter ratio. The width is between 10–50 μm and the thickness is less than 5 μm. The large magnification in the field of view does not show a complete picture of the fibers, but it is certain that most of the individual fibers are at least 500 μm in length. The surface of the WCPF has a large number of hydroxyl groups, which can produce strong hydrogen bonds to make the fibers aggregate, but the fibers can be well separated from each other by beating and grinding. With the increase of beating time, the surface of WCPF becomes rougher and some fibers are teared along the length direction and become finer. The WCPF contains cellulose, hemicellulose, lignin, and a small amount of silicate, and the cellulose has many amorphous regions [18]. The shearing, extrusion, and friction in the beating and grinding process make the fiber surface rougher. The roughness of the fiber surface is conducive to increasing the friction between the WCPF and PLA matrix, and can further improve the mechanical properties of the WCPF/PLA composite [24].

### 3.2. Mechanical Properties of Composites

Table 2 shows the tensile strength, elongation at break, tensile modulus, flexural strength, and flexural modulus of WCPF/PLA composites prepared under different conditions. It can be seen from Table 2 that with the increase of beating time, the tensile strength and flexural strength of WCPF/PLA composites increase first and then decrease. When the beating time of WCPF is 30 min, the tensile strength of the composite with 20 wt% of WCPF reaches the maximum value of 27.7 MPa and the flexural strength is 37.5 MPa. Compared with pure PLA, the tensile strength increases by 14.5%, and the flexural strength increases by 26.3%. When 20 wt% of WCPF with different beating times was added, the elongation at break of the WCPF/PLA composites basically remains stable with a small fluctuation and decreases obviously compared with pure PLA. When the WCPF/PLA composite is stretched, the tensile force can be transferred to WCPF through the interface between WCPF and PLA, and WCPF bears part of the tensile force to improve the tensile strength of the WCPF/PLA composite. The filling of WCPF limits the movement ability of polymer chain, even causing stress concentration, and thus reduces the elongation at break. After beating and grinding, the surface of WCPF becomes rough, which can improve the interfacial binding force between WCPF and PLA matrix via the increase of friction force. However, a too-long beating time leads to the decrease of the mechanical property because the length of WCPF becomes shorter after a long period of shearing and squeezing in the beating process.

The tensile strength of the WCPF/PLA composite increases first and then decreases with the increase of WCPF content from 5 wt% to 30 wt%. When the WCPF content is 25 wt%, the tensile strength of the WCPF/PLA composite reaches the maximum value of 29.5 MPa, which is 21.9% higher than that of pure PLA. The flexural strength shows a gradually increasing trend with the increase of WCPF content. When the WCPF content is 30 wt%, the flexural strength of the WCPF/PLA composite increases to the maximum value of 44.4 MPa, which is 49.5% higher than that of pure PLA. The addition of WCPF can play the role of reinforcing rib in WCPF/PLA composites [19]. With the increase of fiber content, more WCPF inside the WCPF/PLA composite can bear the bending stress thereby improving the flexural strength of the composite.

**Figure 3:**
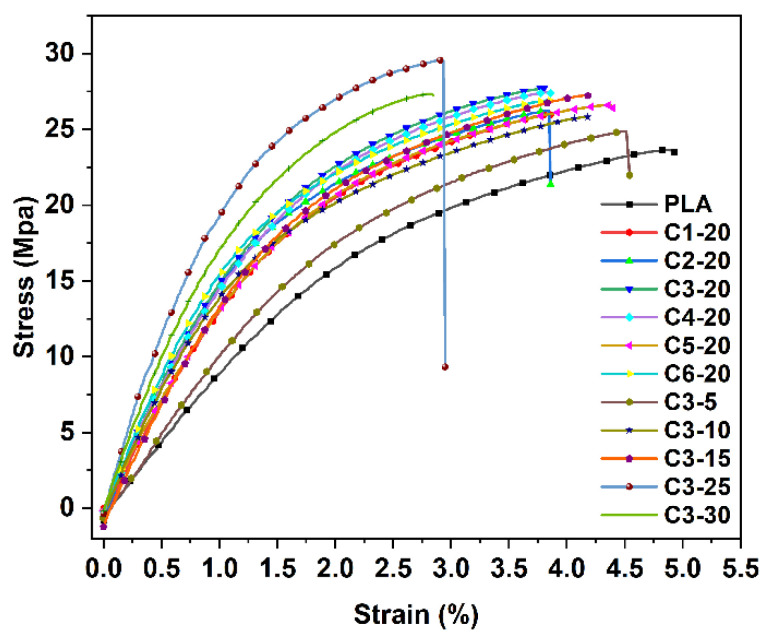
Stress-strain curves of WCPF/PLA composites.

Figure 3 shows the stress–strain curves of WCPF/PLA composites. The tensile and flexural modulus of the WCPF/PLA composites are obviously higher than that of pure PLA and increase with the increase of WCPF content. However, the elongation at break gradually decreases with the increase of WCPF content, indicating that WCPF improves the deformation resistance of the WCPF/PLA composite, but decreases the toughness.

In short, the WCPF can improve the tensile strength, flexural strength, and deformation resistance of the WCPF/PLA composite, but will reduce the toughness. Moreover, excessive fibers will destroy the structure of the polymer itself and cause stress concentration, resulting in a decrease in the tensile strength of the composite.

### 3.3. Morphology of Fracture Surface

**Figure 4:**
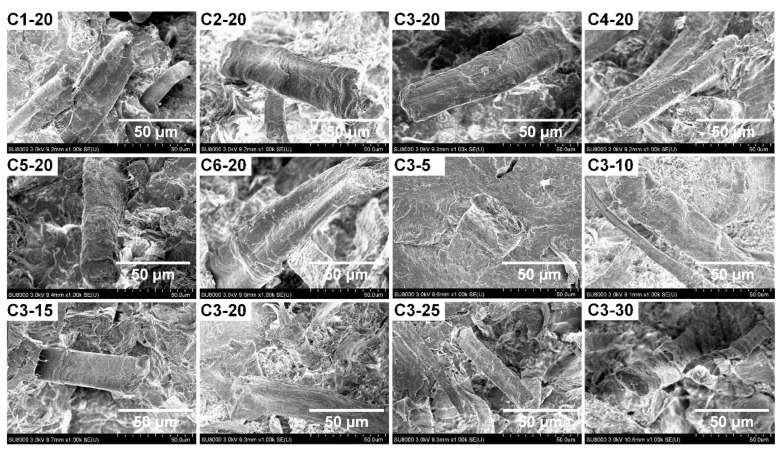
SEM images of fracture surface of WCPF/PLA composites.

Figure 4 shows the SEM images of the fracture surface of WCPF/PLA composites prepared under different conditions. It can be seen that the WCPF are evenly distributed in the PLA matrix, and do not intertwine or reunite with each other. The cross section of the pulled out WCPF changes from a flat shape to an ellipse shape, because the PLA matrix adheres to the WCPF surface. This is because the PLA molecules can impregnate into the crevices of WCPF in the solvent. When the content of WCPF is 5 wt% (C3-5), the WCPF disperses sparsely in the PLA matrix and the enhancement effect is limited. With the increase of the content of WCPF, the density of WCPF increases and still maintains a good dispersion state in the WCPF/PLA composite. When the content of WCPF is 30 wt% (C3-30), some gaps in the PLA matrix can be observed in the fracture surface. This is because the excessive fiber content will destroy the original structure of polymer matrix and reduce the flow index, thus leading to bubble or stress concentration and other defects during processing. In addition, most of the WCPF are pulled out from the PLA matrix instead of breaking and flushing with the section, indicating that the interfacial adhesion between WCPF and PLA matrix is not good. Instead of breaking when subjected to external forces, the fibers are pulled out from the PLA matrix, which limits the improvement effect of mechanical properties.

### 3.4. DSC Analysis

**Figure 5:**
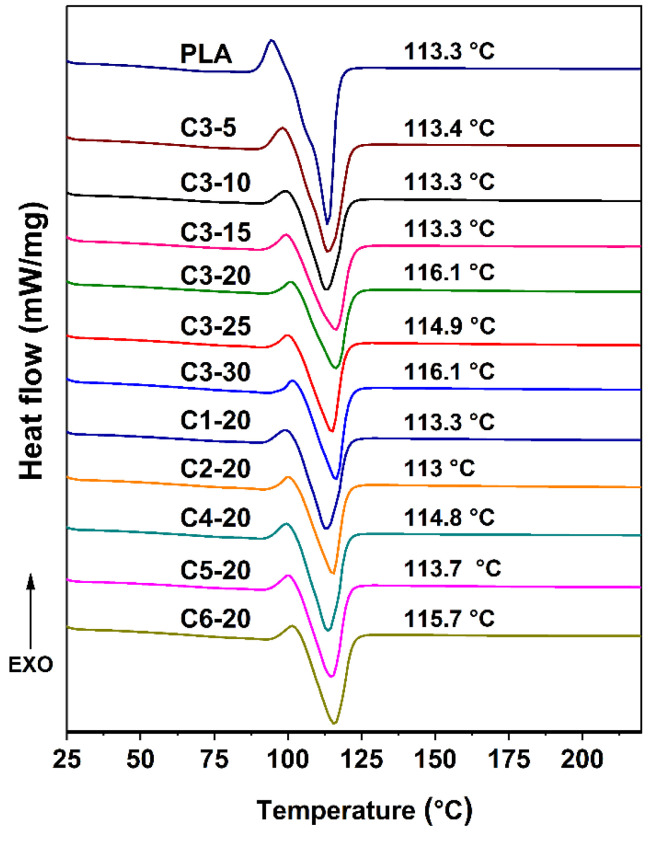
DSC curves of WCPF/PLA composites.

Figure 5 shows the DSC curves of WCPF/PLA composites prepared under different conditions. The WCPF/PLA composite has obvious melting peaks at about 113.3 °C. When the WCPF content is 20 wt%, with the increase of beating time, the melting temperature is basically stable with some small random fluctuations. This is because the WCPF can affect the crystallization of PLA matrix and a trace test sample for the DSC test may lead to the fluctuations of the result [25,26].

With the increase of WCPF content, the melting temperature of the WCPF/PLA composite also remains basically stable and has some random fluctuations. Most of the fluctuations are in the direction of increasing the melting point of the WCPF/PLA composite. This may be due to the change of crystallinity. Moreover, maybe due to the high crystallinity of the raw materials, the glass transition temperature is not observed.

In short, there is no chemical reaction between WCPF and PLA and no new substance is generated, so the beating time and content of WCPF have no obvious effect on the thermal properties of the WCPF/PLA composite.

### 3.5. TG Analysis

**Figure 6:**
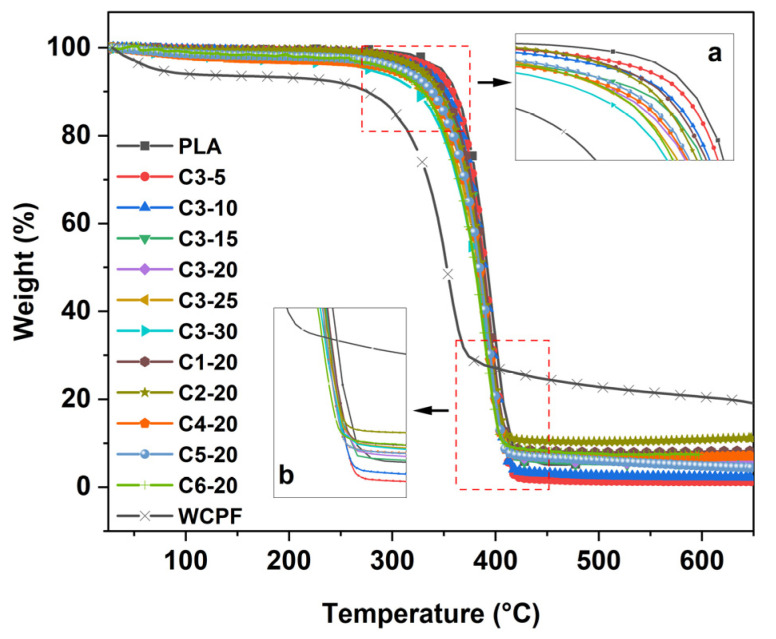
TG curves of WCPF/PLA composites prepared under different conditions. (a) The details of initial weight loss stage, (b) The details of the end of decomposition.

Figure 6 shows the TG curves of WCPF and WCPF/PLA composites prepared under different conditions. The initial thermal decomposition temperature (5 wt% weight loss, the same below) of PLA is about 348 °C and that of the WCPF/PLA composites filled with WCPF generally decrease (280–339 °C) with the increase of WCPF content. When the content of WCPF, with a beating time of 30 min, increases from 5 wt% to 30 wt%, the initial thermal decomposition temperatures of WCPF/PLA composites are 339 °C, 330 °C, 308 °C, 300 °C, 298 °C, and 280 °C, respectively. It can be seen that with the increase of WCPF content, the initial thermal decomposition temperature of the WCPF/PLA composite material gradually decreases. When the beating time increases from 10 min to 60 min with a WCPF content of 20 wt%, the initial thermal decomposition temperature of WCPF/PLA composites are 330 °C, 326 °C, 300 °C, 298 °C, 307 °C, and 298 °C, respectively. With the increase in beating time, the initial thermal decomposition temperature of the WCPF/PLA composites gradually decreases at first and then remains basically stable. This may be because, with the prolongation of beating time, some inorganic fillers and other components in the waste paper are removed more thoroughly.

The initial mass loss is mainly attributed to the moisture volatilization and residual solvents. When the temperature reaches about 250 °C, the hemicellulose, lignin, and cellulose of WCPF begin to decompose, among which the hemicellulose has the lowest thermal stability and can be completely decomposed at about 300 °C. The decomposition temperature of lignin is about 300–400 °C. Cellulose decomposes from about 275 °C to about 420 °C [3,27]. The mass loss after 420 °C is mainly due to the volatilization of coke and other residues from the decomposition of WCPF.

The decomposition temperature of the components in WCPF is lower than that of PLA, so the thermal decomposition temperature of the WCPF/PLA composite is lower than that of the PLA matrix. Therefore, with the increase of WCPF content, the thermal stability becomes worse.

In addition, during the injection molding process of the WCPF/PLA composite, part of the PLA molecular chain structure may be broken, resulting in a decrease in thermal stability.

### 3.6. XRD Analysis

**Figure 7:**
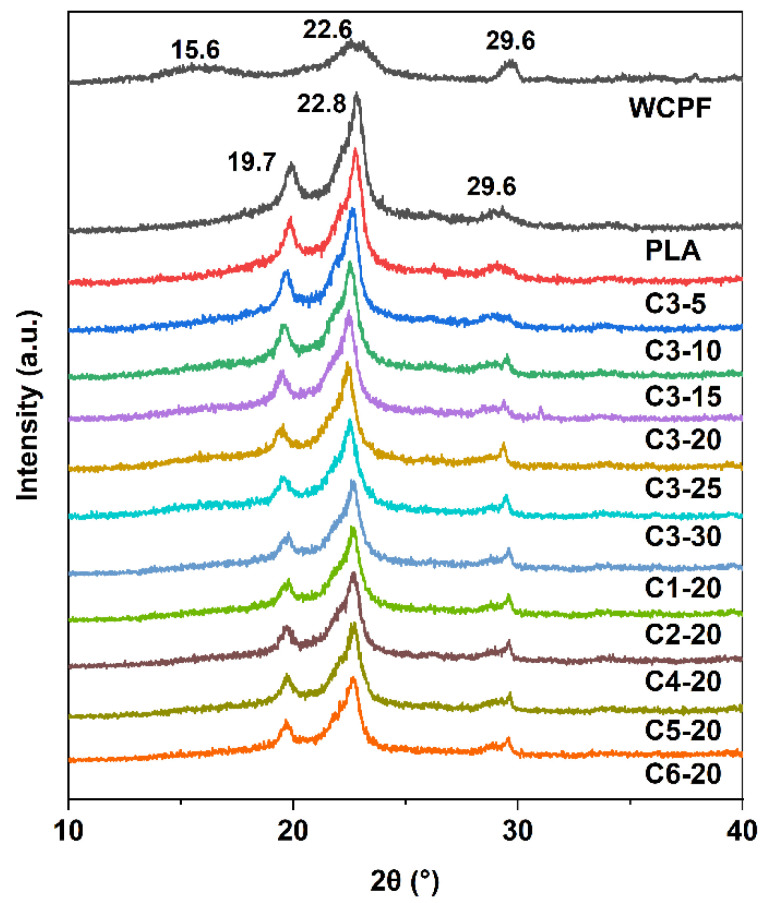
XRD patterns of WCPF and WCPF/PLA composites.

Figure 7 shows the XRD patterns of WCPF and WCPF/PLA composites prepared under different conditions. The diffraction peaks located at 15.6° and 22.6° of WCPF correspond to the crystal planes of (101) and (002) of cellulose. The diffraction peak located at about 29.6° may be due to other unknown components in the WCPF. The three diffraction peaks located at about 19.9°, 22.9°, and 29.6° of PLA and WCPF/PLA composites are the diffraction peaks of the α crystalline PLA matrix [26]. With the increase of WCPF content, the diffraction peaks at 22.9° gradually weakened, and the peaks at 29.6° are obviously strengthened, which is due to the superposition of diffraction peaks of WCPF. The beating time and content of WCPF have no obvious effect on the diffraction patterns. 

With the addition of WCPF, there was no new peak in the diffraction patterns of the WCPF/PLA composite, and the peak position did not change. This is because there is no chemical reaction between WCPF and PLA, and no new material was generated; the beating time and addition of WCPF could not change the crystallographic form of PLA.

## 4. Conclusions

Waste corrugated paper is used as a source of plant fiber. The WCPF is used to improve the mechanical performance of PLA. The results show that WCPF can obviously improve the tensile strength, flexural strength, and deformation resistance of the WCPF/PLA composite. The WCPF/PLA composite at 25 wt% WCPF and with a beating time of 30 min has the best mechanical properties. WCPF can be uniformly dispersed in the PLA matrix by dichloromethane solvent. The addition of WCPF decreases the thermal stability of the WCPF/PLA composite. WCPF has a certain influence on the crystallinity of PLA but does not affect the crystallographic form. The surface modification of the WCPF is worthy of further study to increase the interfacial binding force between WCPF and PLA to improve the reinforcement effect. This work is of great significance for solving the problem of waste paper pollutants and promoting the development of circular economy. In addition, it also provides a new idea for reducing the use cost of polylactic acid and preparing completely environmentally friendly composite materials.

## Figures and Tables

**Table 1:** The different preparation conditions of the WCPF/PLA composite.

Sample Number	Beating Time (min)	Fiber Content (wt%)
PLA	-	0
C1-20	10	20
C2-20	20	20
C3-20	30	20
C4-20	40	20
C5-20	50	20
C6-20	60	20
C3-5	30	5
C3-10	30	10
C3-15	30	15
C3-20	30	20
C3-25	30	25
C3-30	30	30

**Table 2:** Mechanical properties of WCPF/PLA composites with different beating times and content of WCPF.

Samples	Tensile Strength (MPa)	Elongation at Break (%)	Tensile Modulus (MPa)	Flexural Strength (MPa)	Flexural Modulus (MPa)
PLA	24.2 ± 0.50	5.0 ± 0.16	486.3 ± 23.8	29.7 ± 0.13	627.6 ± 3.9
C1-20	25.6 ± 0.29	3.9 ± 0.37	657.0 ± 63.4	35.8 ± 2.59	1245.5 ± 69.5
C2-20	26.3 ± 0.45	3.9 ± 0.45	698.5 ± 64.8	37.4 ± 0.44	1326.4 ± 51.8
C3-20	27.7 ± 0.16	3.8 ± 0.29	717.0 ± 70.8	37.5 ± 0.06	1384.5 ± 44.1
C4-20	27.5 ± 0.24	4.0 ± 0.17	694.6 ± 31.4	38.4 ± 1.16	1366.2 ± 48.5
C5-20	26.6 ± 0.29	3.7 ± 0.14	719.2 ± 29.9	34.7 ± 0.81	1317.8 ± 9.8
C6-20	27.0 ± 0.37	3.8 ± 0.33	709.3 ± 58.9	36.6 ± 0.58	1351.7 ± 58.1
C3-5	24.4 ± 0.80	4.5 ± 0.22	543.0 ± 21.6	31.2 ± 0.69	724.6 ± 24.5
C3-10	25.8 ± 0.57	4.3 ± 0.19	596.5 ± 28.7	31.5 ± 0.63	824.6 ± 14.1
C3-15	27.3 ± 0.33	4.2 ± 0.24	651.2 ± 34.3	35.5 ± 3.25	1128.6 ± 85.8
C3-25	29.5 ± 1.27	3.3 ± 0.21	907.3 ± 80.1	41.8 ± 0.27	1766.2 ± 15.0
C3-30	27.2 ± 0.34	2.9 ± 0.10	948.4 ± 24.1	44.4 ± 0.83	2028.2 ± 8.3

## Data Availability

The authors declare data availability.
